# 2017 Infectious Diseases Society of America Physician Compensation Survey: Results and Analysis

**DOI:** 10.1093/ofid/ofy309

**Published:** 2018-11-22

**Authors:** Robin Trotman, Alice I Kim, Ann T MacIntyre, Jethro Trees Ritter, Anurag N Malani

**Affiliations:** 1 CoxHealth, Springfield, Missouri; 2 Cleveland Clinic, Cleveland, Ohio; 3 Private Practice, Miami, Florida; 4 Central Coast Infectious Disease Consultants, San Luis Obispo, California; 5 St Joseph Mercy Health System, Ann Arbor, Michigan

**Keywords:** compensation, Infectious Diseases, Infectious Diseases Society of America (IDSA), salary, survey

## Abstract

Analyzing health care reimbursement is a dynamic process. Infectious Diseases (ID) physicians have careers in diverse practice models. With current compensation models focusing on value and quality metrics, ID physicians are poised to be at the forefront of these delivery models. Monitoring and disseminating the current status of ID physician compensation are priorities of the Infectious Diseases Society (IDSA). In 2015, the IDSA conducted the largest ID physician compensation survey to date. The data were analyzed and disseminated, and the society subsequently responded with a plan to continue to develop and collect the most comprehensive and accurate data on ID physician compensation. Therefore, from May to June 2017, the IDSA conducted a follow-up compensation survey of its members. This survey resulted in the largest number of respondents of any ID compensation survey. It revealed that compensation across the different practice demographics had increased since the 2015 survey and is generally higher than salaries published in other comparable surveys. These data and the subsequent analyses focus on physicians who report patient care as their primary responsibility; they are presented by members of the IDSA’s Clinical Affairs Committee.

For individuals pursuing careers in medicine, income expectations can significantly influence their choice of specialty. Among medical school students who were interested in Infectious Diseases (ID) but ultimately chose another field, compensation was 1 of 3 main factors that influenced their career decisions [1]. Compensation survey results can be a useful tool to help guide and negotiate salary and fair market value (FMV) for other ID-related work. Accurate descriptions of FMV can be used to directly impact physician salaries and other compensation arrangements.

Previous physician compensation surveys presented data with small samples sizes and did not sufficiently capture the scope and diversity of job opportunities, settings of practice, and/or responsibilities undertaken by ID physicians. ID physicians work within various employment models and in numerous settings, including but not limited to private practices, hospitals, clinics, academic medical centers, and government facilities. Therefore, the previous compensation surveys were limited in their ability to reliably describe the variability in ID physician compensation.

In 2015, the Infectious Diseases Society of America (IDSA) conducted a survey of its members in response to the need for a more comprehensive understanding of ID physician compensation [2]. The primary aim was to capture compensation information from a large sample of respondents working in various settings including clinical care, research, and public health, and thereby to more accurately describe and represent the range of diversity in compensation across the specialty. To further characterize ID physician compensation trends, the IDSA conducted a follow-up survey in 2017. Here, we summarize the results from the 2017 IDSA Compensation Survey, with a focus on ID specialists who indicated patient care as their primary responsibility.

## METHODS

The IDSA conducted a self-administered, web-based, voluntary online survey from May 22 to June 26, 2017. Survey invitations were sent via email to 6793 IDSA physician members, associates, and fellows residing in the United States with an MD, DO, and/or MBBS degree. Students, members-in-training (ie, residents/fellows), and honorary and emeritus members were excluded from the survey. The survey asked a series of quantitative questions and was hosted on a secure website. Respondents were asked to report their income, including bonuses, but excluding income from expert witness testimony and external consultant honoraria. As a response to each question was not mandated, subsequent totals in some areas (such as ethnicity) may not equal 100%. Final reports compiling the 2015 and 2017 survey results are available to IDSA members on the IDSA website (www.idsociety.org) accessed September 1, 2018 in the Manage Your Practice section.

## RESULTS

### Demographics

**Figure 1.**
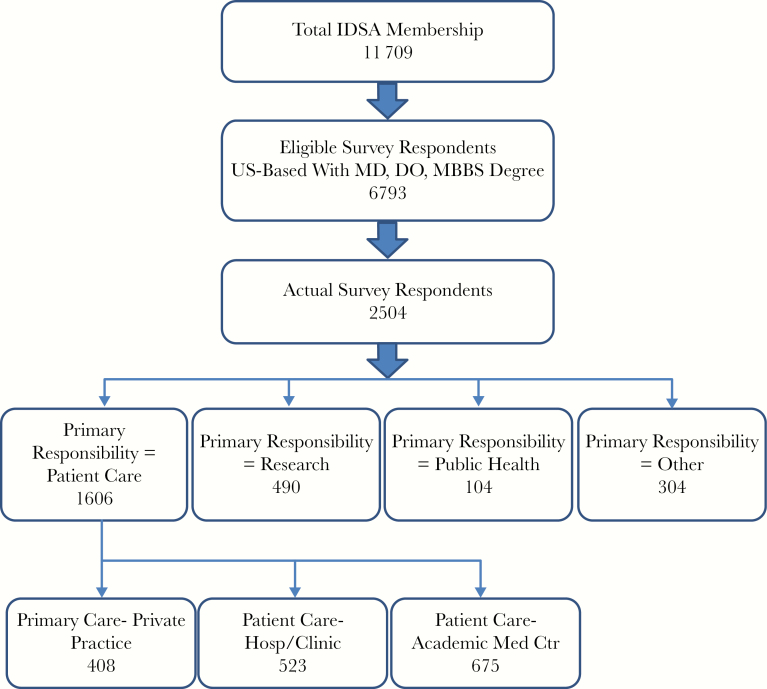
Breakdown of Infectious Diseases Society of America (IDSA) members who responded to the survey.

A total of 2504 US-based respondents completed the 2017 survey (37% response rate). Among the respondents, 40% were female. Ninety percent of all respondents (n = 2243) reported working full time. Responses were well distributed across all regions of the United States. Approximately two-thirds reported patient care as their primary responsibility (64%, n = 1606), followed by research (20%, n = 490) and public health (4%, n = 104), respectively. Within the patient care segment, 25% of respondents reported working in private practice, whereas 42% were employed by an academic medical center and 33% were employed by a hospital or clinic. Figure 1 shows the breakdown of IDSA members who responded to the survey.

The average age of the survey participants was 51 years, and the average years practiced in the ID field was 18 years. Age distributions varied according to employment affiliation. Those employed by a hospital or a clinic had a median age of 46 years, whereas the median age was 53 among those working in public health. The median age of respondents who indicated other employment affiliations (private practice, employment by an academic medical center, or research) was between these 2 medians. Comparison of demographics of the IDSA total membership and the respondents for the compensation survey are shown in Table 1.

**Table 1.** Demographic Comparison Between IDSA Members and Compensation Survey Respondents

	IDSA Members (n = 11 709), %	Compensation Survey Respondents (n = 2504), %
Gender		
Male	59	59
Female	41	40
Ethnicity		
Asian	21	14
Black/African American	4	3
Hispanic	8	8
White/Caucasian	67	60
Age, y		
<40	29	20
40–49	24	30
50–59	19	22
60+	28	26
Primary responsibility		
Patient care (overall)	61	65
Research & teaching	24	20
Public health	4	4
Other^a^	11	11

Abbreviation: IDSA, Infectious Diseases Society of America.

^a^Other: administration, hospital epidemiology, or other.

### Analysis of Compensation in the Patient Care Segment

Among all subcategories of respondents who indicated patient care as their primary responsibility, large majorities reported working full-time, including participants working in private practice (90%), those employed by a hospital or clinic (90%), and those employed by an academic medical center (94%). The percentage of respondents who reported full-time work also was high in the research (94%) and public health categories (86%). This analysis and the income figures are based solely on data from respondents who reported working full-time with a primary focus on patient care (Table 2). Income results based on fewer than 10 answering respondents in a category were excluded to ensure respondent confidentiality.

**Table 2.** Overall Compensation Results

Membership Segment	Total Sample	% Female	% Full-time	Average Compensation (Full-time)	Median Compensation (Full-time)	25th Percentile	75th Percentile	90th Percentile
Overall	2504	40	90	238 300	212 200	165 000	275 000	350 000
Patient care	1606	40	92	240 800	215 000	170 000	275 000	350 000
Research	490	42	94	211 500	181 500	150 000	254 800	319 000
Public health	104	50	86	188 600	189 500	160 000	219 500	243 300
Other^a^	304	N/A	72	300 200	265 000	200 000	361 000	450 000

^a^Other: administration, hospital epidemiology, or other.

In general, full-time ID physicians in private practice (n = 366) reported higher incomes, with a median annual salary of $260 000, than respondents employed by hospitals, clinics, or academic medical centers (median salaries of $237 500 and $181 500, respectively) (Table 3). The highest median salary ($300 000) was among those who indicated they were a solo practitioner or owner. Physicians employed within a private practice as an associate had a median salary of $208 000. Average annual salaries were higher than median figures among respondents, but both measures revealed similar relative differences between solo practitioners and owners compared with employed associates. Overall, individuals in private practice reported an average annual compensation of $316 600.

**Table 3.** Compensation for Patient Care

Patient Care Segment	Total Sample	% Female	% Full-time	Average Compensation (Full-time)	Median Compensation (Full-time)	25th Percentile	75th Percentile	90th Percentile
Overall patient care	1606	40	92	240 800	215 000	170 000	275 000	350 000
Private practice	408	32	90	316 600	260 000	194 000	359 300	511 300
Solo/owner/partner	291	26	91	344 400	300 000	157 200	-	586 000
Associate	117	38	84	238 700	208 000	152 000	-	400 000
Hospital/clinic-employed	523	40	90	248 700	237 500	200 000	280 000	350 000
AMC-employed	675	47	94	192 600	181 500	150 000	221 700	271 200

Abbreviation: AMC, academic medical center.

The majority of hospital- or clinic-employed physicians (n = 472) provided care for most of their patients in the inpatient hospital setting. Their median income was $250 000, which was higher than similarly employed physicians who provided care for most of their patients in a hospital-based ambulatory clinic or a community-based clinic. Similar differences in compensation were seen among physicians employed by academic medical centers (n = 636). Among these respondents, the median annual salary was $181 500, which represented the lowest compensation among survey participants in the patient care segment (Table 3). Of these physicians, 37% reported having an academic administrative appointment, and their median salary was $40 000 higher than the median salary of those without an administrative appointment.

Among all survey participants working primarily in patient care, regardless of employment affiliation or facility type, reported income for women was significantly lower than that for men, with the exception of women who were employed by academic medical centers early in their careers (Table 4). Additional analysis of the survey results that is focused on this important income disparity in the ID subspecialty is underway.

**Table 4.** Average Compensation for Patient Care, Full-time Respondents by Age and Gender

	Age <40 y		Age 40–49 y		Age 50–59 y		Age ≥60 y	
	Male	Female	Male	Female	Male	Female	Male	Female
PP: sole owner/partner/solo	276 600	231 200	373 300	328 400	383 900	305 500	346 500	301 200
PP: associate/employee	220 500	186 100	290 100	224 900	-	-	229 700	-
Hospital/clinic-employed	233 700	192 000	270 100	227 300	293 600	247 300	263 800	240 000
AMC-employed	163 000	157 900	191 800	179 900	239 200	194 200	244 100	203 500

Abbreviations: AMC, academic medical center; PP, private practice.

## DISCUSSION

Promoting the value of ID specialists is 1 of the IDSA’s 6 strategic priorities [3]. Within the society’s governance structure, The Clinical Affairs Committee is the entity that produces resources and programming to support this strategic priority. This group is comprised of volunteer physicians passionate about positioning ID within the evolving health care landscape and working to ensure that ID physicians are compensated appropriately for the diverse and invaluable services they provide. In doing so, it is of paramount importance that we understand and quantify the value of cognitive vs procedure-based medical care. The IDSA is also charged with the identification and further delineation of compensation for non–patient care activities (ie, antimicrobial stewardship, infection prevention, other hospital administrative duties, etc). These roles become increasingly important as physicians migrate to or enter into practice in employed or large group models. Furthermore, with the development of value-based and shared-risk models, ID physicians are poised to add value to and lead many of these initiatives. Quantifying the value that ID physicians provide requires accurate estimations of current ID physician compensation. Therefore, the IDSA has committed to developing the most comprehensive and granular compensation assessments and to making these data readily accessible and usable.

This 2017 IDSA compensation survey provides an up-to-date and detailed picture of income earned across the wide-ranging career activities that ID physicians can pursue, with a specific focus on those IDSA members who select patient care (ie, clinical practice) as their primary responsibility. The survey captures the broad array of different employment arrangements and practice affiliations that apply to ID physicians working across the health care continuum. These data are highly relevant to those considering careers in Infectious Diseases, as well as practicing ID specialists, employers, and administrators. The demographics assessed in the survey are representative of the IDSA membership and the ID specialty at large (Table 1). Historically, other sources reporting ID compensation, such as the Medical Group Management Association (MGMA) or Medscape [4], have relied on smaller sample sizes that are not always representative of the entire specialty or the current trends in compensation. In this 2017 IDSA survey, the number of respondents in just the full-time, employed patient care segment alone is 2–3 times larger than the complete data set from any other ID physician compensation survey. In addition, we provide salary averages and median compensation to address accuracy concerns with other surveys. For instance, average salary can be influenced by extremely large or small values.

The 2017 Medscape Physician Compensation Report and Society of Hospital Medicine’s survey were based on a sample of approximately 385 and 528 ID physicians, respectively [4, 5]. The numbers of ID physicians included in both the 2017 (n = 2504) and 2015 (n = 1878) IDSA surveys were significantly greater and represent the largest sample sizes of any currently available survey of ID compensation.

The average annual compensation (median salary was not included) reported in Medscape’s report for ID specialists was $228 000, which was described as a 6% increase from the previous year’s survey [4]. In the Society of Hospital Medicine’s Survey, the average annual compensation for ID physicians was reported to be $283 191 [5], and this average salary was 8% higher than the average ID salary ($261 791) reported in that organization’s previous survey in 2015 [5]. Compared with results from the IDSA compensation survey from 2015, both the median and average income levels in the 2017 survey were somewhat higher. For example, the median salary for all respondents working full-time in private practice was $260 000 in 2017, compared with $248 000 in 2015.

The IDSA’s surveys from 2015 and 2017 capture the largest sample sizes studying the compensation data of ID physicians in the various employment settings and affiliations (eg, private practice, hospital employment, public health), with the most recent focus on the analyses of physicians working in the patient care segment. The number of respondents and the granularity of the data suggest that these survey results are the most authoritative sources of information depicting ID specialists’ compensation and therefore should be incorporated into FMV analyses, especially those generated by health care consulting firms. Employers may use these analyses to negotiate physician salaries. Often the surveys referred to in these analyses are small and may not represent data from comparable practice scenarios. Subsequently, the IDSA has been able to capture a large yet detailed data set of ID physician compensation in various employment models in an effort to use the report for FMV analyses, contract negotiations, and employment opportunities.

The IDSA is dedicated to pursuing efforts to correlate ID physician compensation with the value provided by ID physicians in the realms of patient care, health services management, and public health. In this pursuit, the IDSA feels that it is imperative to address misleading perceptions of income trends. These perceptions of compensation are increasingly relevant, especially as they pertain to the ID workforce. Medical student debt has risen dramatically in recent years. This is especially important to trainees pursuing careers in medicine who might perceive the ID specialty as relatively low-paying when compared with other specialties. When graduating internal medicine residents were asked what intervention would increase their interest in ID, the most common response was improving the salary of ID physicians [6]. Subsequently, trainees have become more attracted to higher-paying specialties, which historically have been procedure-based disciplines. In order for the specialty of ID to continue to grow and thrive, we must effectively communicate to medical students, residents, and others in the health care system the broader picture of what ID physicians do and what they earn. We must continue to convey the value that ID care provides to the health care system at large, both to the individual and populations of patients. As with the 2015 compensation survey, these data reveal similar gender income disparities. This is a finding that will be specifically addressed in future surveys, and there are further analyses and discussions on the gender income disparity underway. We hope that this and subsequent compensation surveys provide a more accurate picture of the potential income earned in the field of ID and raise awareness regarding the actual compensation trends within our specialty. The IDSA continues to foster these efforts with plans for a follow-up compensation study in 2019.

## CONCLUSIONS

Compensation can be an important factor in choosing specialty training. For recruitment and retention in the field of Infectious Diseases, clarity about financial incentives is of increasing importance. Previous studies have been inadequate due to a low number of respondents. This current 2017 compensation study is the most representative of ID physicians who focus on patient care across different ID practice and employment affiliations. Compared with results from the IDSA compensation survey from 2015, both the median and average income levels in this most recent survey were somewhat higher, with the median salary for all respondents working full-time in private practice increasing from $248 000 to $260 000.

We hope the findings of this report will be used to implement strategies that will result in attracting the best and brightest to our field and assist in employment choice and negotiations. Gender income disparity and geographic variations in compensation are areas for future study.
